# Hormonal responses upon return of spontaneous circulation after cardiac arrest: a retrospective cohort study

**DOI:** 10.1186/cc10019

**Published:** 2011-02-07

**Authors:** Jin Joo Kim, Sung Youl Hyun, Seong Youn Hwang, Young Bo Jung, Jong Hwan Shin, Yong Su Lim, Jin Seong Cho, Hyuk Jun Yang, Gun Lee

**Affiliations:** 1 Department of Emergency Medicine, Gachon University Gil Hospital, 1198 Guwoldong Namdonggu Incheon, 405-760, South Korea; 2 Department of Cardiovascular Surgery, Gachon University Gil Hospital, 1198 Guwoldong Namdonggu Incheon, 405-760, South Korea; 3 Department of Emergency Medicine, Sungkunkwan University School of Medicine, Samsung Changwon Hospital, 50 Hapseongdong MansanHoiwongu, Changwon, 630-520, South Korea; 4 Department of Emergency Medicine, Seoul Metropolitan Government Seoul National University Boramae Medical Center, 40 Boramaegil, Dongjakgu, Seoul, 156-707, South Korea

## Abstract

**Introduction:**

Cardiac arrest is often fatal and can be extremely stressful to patients, even if spontaneous rhythm is returned. The purpose of this study was to analyze the hormonal response after return of spontaneous circulation (ROSC).

**Methods:**

This is a retrospective review of the chart and laboratory findings in a single medical facility. The patients admitted to the intensive care unit after successful resuscitation after out-of-hospital cardiac arrest were retrospectively identified and evaluated. Patients with hormonal diseases, patients who received cortisol treatment, those experiencing trauma, and pregnant women were excluded. Serum cortisol, adrenocorticotropic hormone (ACTH), and anti-diuretic hormone (ADH (vasopressin)) were analyzed and a corticotropin-stimulation test was performed. Mortality at one week and one month after admission, and neurologic outcome (cerebral performance category (CPC)) one month after admission were evaluated.

**Results:**

A total of 117 patients, including 84 males (71.8%), were evaluated in this study. One week and one month after admission, 87 (74.4%) and 65 patients (55.6%) survived, respectively. Relative adrenal insufficiency, and higher plasma ACTH and ADH levels were associated with shock-related mortality (*P *= 0.046, 0.005, and 0.037, respectively), and ACTH and ADH levels were also associated with late mortality (*P *= 0.002 and 0.004, respectively). Patients with relative adrenal insufficiency, ACTH ≧5 pg/mL, and ADH ≧30 pg/mL, had a two-fold increased risk of a poor outcome (shock-related mortality): (odds ratio (OR), 2.601 and 95% confidence interval (CI), 1.015 to 6.664; OR, 2.759 and 95% CI, 1.060 to 7.185; OR, 2.576 and 95% CI, 1.051 to 6.313, respectively). Thirty-five patients (29.9%) had a good CPC (1 to 2), and 82 patients (70.1%) had a bad CPC (3 to 5). Age ≧50 years and an ADH ≧30 pg/mL were associated with a bad CPC (OR, 4.564 and 95% CI, 1.794 to 11.612; OR, 6.568 and 95% CI, 1.918 to 22.483, respectively).

**Conclusions:**

The patients with relative adrenal insufficiency and higher blood levels of ACTH and ADH upon ROSC after cardiac arrest had a poor outcome. The effectiveness of administration of cortisol and ADH to patients upon ROSC after cardiac arrest is uncertain and additional studies are needed.

## Introduction

The recovery of spontaneous circulation (ROSC) after cardiac arrest results in a whole body ischemia-reperfusion syndrome called 'post-cardiac arrest syndrome' [1,2]. Post-cardiac arrest syndrome is a unique pathophysiological process that involves multiple organs including post-cardiac arrest brain injury, post-cardiac myocardial dysfunction, systemic ischemia/reperfusion response and persistent precipitating pathophysiology [1]. Post-cardiac arrest syndrome resembles multiorgan failure or septic shock but with a more comprehensive meaning. Immediately after ROSC, the heart rate and blood pressure are very unstable. Most patients need inotropics and vasopressors to manage hypotension due to impaired vasoregulation, myocardial dysfunction, and volume depletion. Also, the possibility of multi-organ failure and infection is increased because of systemic inflammatory immune responses and activation of the coagulation cascade [3,4]. In this study, several serum hormonal concentrations, including cortisol, adrenocorticotropic hormone (ACTH), and anti-diuretic hormone (ADH (vasopressin)) were analyzed in relation to mortality and neurologic outcome upon ROSC after cardiac arrest.

## Materials and methods

### Study population

The study institution is a 1,300-bed university hospital with an annual emergency intensive care unit (EICU) census of 1,000. This was a retrospective study of patients with ROSC (>24 hours) after cardiac arrest who were admitted to the EICU over a 34-month period between March 2007 and December 2009. The basal characteristics of the patients and the hormonal concentrations (cortisol, ACTH, and ADH) were obtained from laboratory results in their medical records. The patients with underlying hormonal disease (adrenal insufficiency and diabetes insipidus), patients who had already received cortisol and ADH, patients who died within 24 hours of admission, patients who received trauma and patients who were pregnant were excluded. This study was approved by the institutional review board of our center. Informed consent of blood sampling and study was obtained from the next of kin of the patients following the protocol used in our department.

### Clinical evaluation and outcomes

The basal characteristics of patients and outcomes were evaluated. Mortality and neurologic outcomes (Cerebral Performance Category (CPC)) were evaluated. We divided the patients into the following three groups: 1) survivors and non-survivors one week after admission (shock-related mortality); 2) survivors and non-survivors one month after admission (late dead from neurological dysfunction including brain death or cardiovascular problem including myocardial infarction and so on); and 3) good CPC (1, 2) and poor CPC (3 to 5) one month after admission.

### Laboratory variables

The serum cortisol, ACTH, and ADH levels were measured on the first morning after admission to the EICU (between 12 and 24 hours after ROSC). Tetracosactrin (250 μg, Synacthene^®^, NORVATIS, Australia) was administered intravenously and blood samples were obtained immediately before injection, and 30 and 60 minutes after injection. The hormonal concentrations were measured by chemiluminescence immunoassay (CLIA). Relative adrenal insufficiency was defined as an increase in serum cortisol of ≤9 μg/dL.

### Statistical analysis

The data were analyzed using SPSS software (version 16.0; SPSS, Inc., Chicago, IL, USA). A t-test and chi-square test were used and single and multiple variable logistic regression model analyses were performed to estimate the odds ratios of dying, along with 95% confidence intervals (CIs). Statistical significance was defined as a *P-*value < 0.05.

## Results

The basal characteristics of the patients are shown in Table 1. A total of 117 patients were evaluated in this study; there were 84 males (71.8%). Forty-nine (41.9%) patients had relative adrenal insufficiency on the first morning after admission to the EICU. One week and one month after admission, 87 (74.4%) and 65 patients (55.6%) survived, respectively. Thirty-five patients (29.9%) had a good CPC.

**Table 1:** Basal characteristics of the patients (N = 117, median (IQR))

Gender, M, n (%)	84 (71.8)
Age (yr)	52(44 to 64)
BLS time (minutes)	5 (2 to 10)
Arrest time (minutes)	36 (21 to 51)
Epinephrine (mg)	3 (2 to 8)
HTN, yes, n (%)	33 (28.2)
DM, yes, n (%)	13 (11.1)
APACHE II	23 (20 to 27)
SOFA	10 (8 to 11)
Lactate (mmol/L)	8.7 (6.6 to 11.8)
Cortisol, basal (μg/dL)	24.91 (15.00 to 37.36)
Cortisol, 30 minutes (μg/dL)	28.73 (22.71 to 40.19)
Cortisol, 60 minutes (μg/dL)	29.56 (23.49 to 42.76)
RAI, Yes, n (%)	49 (41.9%)
ACTH (pg/mL)	5.40 (1.24 to 23.94)
ADH (pg/mL)	22.11 (12.42 to 35.19)
Survivors, at 1 wk	87 (74.4%)
Survivors, at 1 mo	65 (55.6%)
CPC, good, n (%)	35 (29.9%)

ACTH, adrenocorticotropic hormone; ADH, antidiuretic hormone, vasopressin; APACHE, Acute Physiology and Chronic Health Evaluation; BLS, basic life support; CPC, cerebral performance category (good CPC: 1 to 2, poor CPC: 3 to 5).

DM, diabetes mellitus; HTN, hypertension; SOFA, The Sequential Organ Failure Assessment; RAI, relative adrenal insufficiency.

The hormonal concentrations by mortality and neurologic outcomes are shown in Table 2. The basal cortisol concentration was not significantly related to mortality one week and one month after admission, or CPC score, but the ACTH and ADH concentrations were significantly related to mortality one week and one month after admission, and the CPC score (Table 2).

**Table 2:** Variables by RAI, mortality, and neurologic outcome (N = 117, median values)

	RAI			1 wk mortality			1 mo mortality			CPC		
				
	Yes	No	*P-v*alue	Survived	Dead	*P-*value	Survived	Dead	*P-*value	Good	Poor	*P-*value
	(n = 68)	(n = 49)		(n = 87)	(n = 30)		(n = 65)	(n = 52)		(n = 35)	(n = 82)	
Gender, M, n (%)	49 (72.1)	35 (71.4)	0.940	62 (71.3)	22 (73.3)	0.828	49 (75.4)	35 (67.3)	0.335	31 (88.6)	53 (64.6)	0.008*
Age (yr)	52	53	0.772	52	54	0.422	49	56	0.174	47	56	0.010*
BLS time (minutes)	6	4	0.076	5	6	0.093	5	6	0.207	4	6	0.200
Arrest time (minutes)	40	27	0.004*	31	50	<0.001*	25	44	<0.001*	25	38	0.010*
Epinephrine (mg)	5	2	<0.001*	3	7	0.001*	3	5	0.004*	3	4	0.338
HTN, yes, n (%)	18 (26.5)	15 30.6)	0.507	22 (25.3)	11 (36.7)	0.194	18 (27.7)	15 (28.8)	0.804	9 (25.7)	24 (29.3)	0.668
DM, yes, n (%)	6 (8.8)	7 (14.3)	0.283	8 (9.2)	5 (16.7)	0.298	5 (7.7)	8 (15.4)	0.152	1 (2.9)	12 (14.6)	0.102
APACHE II	23	22	0.889	22	27	0.001*	22	26	<0.001*	31	26	<0.001*
SOFA	10	9	0.062	9	12	<0.001*	9	11	0.001*	9	10	0.029*
Lactate (mmol/L)	9.40	7.55	0.013*	8.50	9.40	0.244	8.40	9.35	0.190	8.30	8.90	0.393
Cortisol, basal (μg/dL)	27.33	16.70	<0.001*	25.05	24.10	0.762	22.74	25.57	0.204	20.45	25.95	0.030*
Cortisol, 30 minutes (μg/dL)	28.74	28.54	0.543	28.89	27.35	0.380	28.29	29.54	0.584	26.45	29.32	0.273
Cortisol, 60 minutes (μg/dL)	29.12	32.46	0.100	29.67	24.64	0.154	29.20	30.74	0.946	29.02	31.53	0.572
ACTH (μg/dL)	7.85	2.12	0.006*	2.60	11.79	0.005*	2.35	9.41	0.002*	2.31	7.85	0.014*
ADH (pg/mL)	24.97	17.11	0.031*	19.81	28.65	0.037*	18.16	28.27	0.004*	14.42	24.80	0.001*

*P < 0.05

ACTH, adrenocorticotropic hormone; ADH, antidiuretic hormone, vasopressin; APACHE, Acute Physiology and Chronic Health Evaluation; BLS, basic life support; CPC, cerebral performance category (good CPC: 1 to 2, poor CPC: 3 to 5); DM, diabetes mellitus; HTN, hypertension; RAI, relative adrenal insufficiency; SOFA, The Sequential Organ Failure Assessment.

Multiple logistic regression by mortality and CPC are shown in Tables 3, 4 and 5. Relative adrenal insufficiency was significantly related to mortality one week after admission by multiple logistic regression analysis (odds ratio (OR), 2.601 and 95% confidence interval (CI), 1.015 to 6.664), but not significantly related to mortality one month after admission and CPC. ACTH was related to mortality one week after admission, but not to others. ADH was related to mortality one week and one month after admission, and CPC based on multiple logistic regression analysis.

**Table 3:** Multiple logistic regression for mortality (at one week after admission)

Factors	*P-*value	Odds ratio	95% CI of odds ratio		Observed power
				
			Lower	Upper	
Age ≧50 yr	0.221	1.784	0.705	4.510	0.261
RAI (+)	0.046	2.601	1.015	6.664	0.371
ACTH ≧5 pg/mL	0.038	2.759	1.060	7.185	0.428
ADH ≧30 pg/mL	0.038	2.576	1.051	6.313	0.343

ACTH, adrenocorticotropic hormone; ADH, antidiuretic hormone; RAI, relative adrenal insufficiency.

**Table 4:** Multiple logistic regression for mortality (at one month after admission)

Factors	*P-*value	Odds ratio	95% CI of odds ratio		Observed power
				
			Lower	Upper	
Age ≧50 yr	0.008	3.217	1.348	7.680	0.770
ACTH ≧5 pg/mL	0.054	2.265	0.987	5.198	0.499
ADH ≧30 pg/mL	0.008	3.463	1.389	8.635	0.787

ACTH, adrenocorticotropic hormone; ADH, antidiuretic hormone.

**Table 5:** Multiple logistic regression for bad CPC (at one month after admission)

Factors	*P-*value	Odds ratio	95% CI of odds ratio		Observed power
				
			Lower	Upper	
Age ≧50 yr	0.001	4.564	1.794	11.612	0.914
ACTH ≧5 pg/mL	0.110	2.125	0.843	5.356	0.346
ADH ≧30 pg/mL	0.003	6.568	1.918	22.483	0.891

ACTH, adrenocorticotropic hormone; ADH, antidiuretic hormone; CPC, Cerebral performance category.

## Discussion

Cardiac arrest is an extremely stressful condition, but few studies have investigated the hormonal response after a cardiac arrest. Post-cardiac arrest syndrome shares many features with severe sepsis or shock, including plasma cytokine elevation with deregulated cytokine production, the presence of endotoxin in plasma, coagulation abnormalities, and adrenal dysfunction [1,5]. Post-cardiac arrest syndrome is a mixed condition of systemic inflammatory responses, hypovolemia, and myocardial dysfunction. During severe illness, including cardiac arrest, many factors can impair the normal hormonal response affecting the hypothalamic-pituitary-adrenal axis [6].

ADH is essential for cardiovascular homeostasis and release from the hypothalamus [7]. ADH synthesis is primarily mediated by variations in plasma effective osmolality and in blood volume or pressure. Landry *et al. *[8] reported that patients in septic shock indicated inappropriately low plasma ADH levels and an increased pressor sensitivity to exogenous ADH. Relative ADH deficiency may contribute to vasodilatory shock in sepsis, likely because of impaired baroreflex-mediated hormone secretion [9,10]. Sharshar *et al. *[11] determined the serum ADH levels in patients with septic shock; serial plasma ADH levels were obtained at baseline, and 6, 24, 48, and 96 hours after the onset of shock. The study showed that plasma ADH levels are increased in nearly all cases during the initial phase of septic shock, and decreased thereafter. A relative ADH deficiency is more likely to occur 36 hours from the onset of shock [11]. Thus, the time at which ADH is measured in relation to the onset of shock is important. In the current study, the hormonal concentrations were measured on the first morning after ICU admission (12 to 24 hours after ROSC). The optimal timing for obtaining the basal and post-stimulation serum cortisol concentrations was recommended at that time, and this was helpful in estimating a prognosis of post-resuscitation disease [12]. Jochberger *et al. *[13] reported that serum ADH concentrations in patients after cardiac surgery (n = 96; 19.5 ± 30.4 pg/mL) were significantly higher than patients with sepsis (n = 25; 6.5 ± 4.3 pg/mL), and patients admitted for non-surgical diseases (n = 51; 6.5 ± 4.3 pg/mL; *P *< 0.001). Leclere *et al. *[14] reported that the median ADH concentration in children with meningococcal septic shock was 41.6 pg/mL and was higher in non-survivors, but not significantly higher. In the current study, we divided the patients into two groups based on the ADH concentration. An ADH concentration >30 pg/mL was associated with a two- to three-fold increased risk of bad outcomes (early and late deaths or poor CPC) based on multiple logistic regression analysis. The basal cortisol concentration was increased initially and not significantly associated with mortality and CPC, but relative adrenal insufficiency was related to early death (within one week following admission). The patients with an ACTH level >5 pg/mL had a three-fold increased risk of early death. Based on the results of the current study, we suggest that within 24 hours of ROSC after cardiac arrest, the cortisol, ACTH, and ADH levels are immediately secreted from hypothalamic-pituitary-adrenal activation following an extremely stressful condition. An adequate initial endogenous stress response may correlate with recovery or even subsequent survival [13]. Hekimian *et al. *[15] reported that patients who die of early refractory shock after cardiopulmonary resuscitation may have an inadequate adrenal response to the stress. Relative adrenal insufficiency was related to poor prognosis or increased mortality rate in patients with resuscitation after cardiac arrest [12,16]. The patients with relatively rapid onset adrenal insufficiency, higher plasma ACTH and ADH levels are related to death, and high concentrations of ADH are also related to bad neurologic outcomes. Because these patients have high concentrations of hormones already, the replacement of hormones, including cortisol and vasopressin, is uncertain. ADH did not reduce the mortality rates as compared with other drugs among patients with septic shock, and ADH did not have a benefit over other drugs in increasing survival to discharge or improving neurologic outcomes in patients after cardiac arrest [17,18].

Our study had the following limitations. First, this was a retrospective, one-center study. Second, the sample sizes were small. Third, hormonal responses are very complicated. Variable factors could affect the hormonal responses in our study.

## Conclusions

The patients with relative adrenal insufficiency and higher blood levels of ACTH and ADH upon ROSC after cardiac arrest had a poor outcome. The effectiveness of administration of cortisol and ADH to patients upon ROSC after cardiac arrest is uncertain; additional studies are needed.

## Key messages

• The axis of the hypothalamic-pituitary-adrenal gland was activated in patients with ROSC after cardiac arrest.

• The patients with relative adrenal insufficiency, still higher ACTH and ADH had a poor outcome.

## Abbreviations

ACTH: adrenocorticotropic hormone; ADH: antidiuretic hormone, vasopressin; APACHE: Acute Physiology and Chronic Health Evaluation; BLS: basic life support; CLIA: chemiluminescence immunoassay; CPC: cerebral performance category (good CPC: 1 to 2, poor CPC: 3 to 5); DM: diabetes mellitus; EICU: emergency intensive care unit; HTN: hypertension; OR: odds ratio; RAI: relative adrenal insufficiency; ROSC: return of spontaneous circulation; SOFA: Sequential Organ Failure Assessment.

## Competing interests

The authors declare that they have no competing interests.

## Authors' contributions

All authors took part in the work and agree with the contents of the manuscript. JJK, SYH, JHS and GL were involved in study conception and design, JJK, HJY and YSL collected clinical data. YBJ, JSC and SYH performed the data analysis. JJK and SYH drafted the manuscript. All authors read and approved the final version of the manuscript.
